# Conceiving during the first postoperative year after bariatric surgery: a retrospective study of pregnancy outcomes

**DOI:** 10.1186/s12884-024-07047-0

**Published:** 2024-12-26

**Authors:** Sesilia Kaukonen, Susanna Pajula, Mika Gissler, Anne Juuti, Veli-Matti Ulander, Marja Kaijomaa

**Affiliations:** 1 Department of Obstetrics and Gynecology, Helsinki University Women’s Hospital, Haartmaninkatu 2, Helsinki, 00029 Finland; 2 Department of Plastic and General Surgery, Turku University Hospital, Turku, Finland; 3 Department of Data and Analytics, Finnish Institute for Health and Welfare, Helsinki, Finland; 4 Department of Molecular Medicine and Surgery, Karolinska Institute, Stockholm, Sweden; 5 Abdominal Center, Department of Abdominal Surgery, Helsinki University Hospital, Helsinki, Finland

**Keywords:** Obesity, Bariatric surgery, Surgery-to-conception-time, Pregnancy outcome, Preterm delivery, Newborn outcome, Low birth weight, Small for gestational age

## Abstract

**Background:**

An increasing number of childbearing-aged women have undergone bariatric surgery (BS). Although pregnancy outcomes generally improve after BS, concern remains over the impact of rapid weight loss and the catabolic state that occurs soon after BS. At least a 12-month surgery-to-conception time (SCT) is recommended, though the reasoning behind this has been questioned. This retrospective study was conducted to compare post-BS pregnancies with SCTs of less (Group 1) or more (Group 2) than 12 months.

**Methods:**

The Hospital Register and Finnish Medical Birth Register were queried for data on BS and subsequent pregnancies. The characteristics of women at surgery and maternal and newborn outcomes in post-BS pregnancies were collected.

**Results:**

Between 2010 and 2022, 113 women gave birth after BS. This included 17 and 96 patients in Groups 1 and 2. The mean SCTs were 8.0 ± 2.7 and 39.0 ± 24.3 months (*p* < 0.001), respectively. At BS, the characteristics of age (*p* = 0.316), weight (*p* = 0.718), body mass index (BMI) (*p* = 0.114) and surgical technique used (*p* = 0.648) were similar. During pregnancy, the mean age of Group 1 women was significantly lower (*p* = 0.005). With no difference in weight (*p* = 0.961) or BMI (*p* = 0.567), the incidence of gestational diabetes mellitus (GDM, *p* = 0.039) was higher in Group 2. The mean gestational age at delivery (*p* = 0.206) and incidence rates of preterm (*p* = 0.645), post-term (*p* = 1.00) and caesarean deliveries (*p* = 1.00) in the groups were similar. A significantly higher incidence of delivery induction (*p* < 0.001 was detected in Group 2. The mean newborn weight in Group 1 was lower (*p* = 0.038), but the mean birth weight standard deviation (*p* = 0.883) and incidences of low birth weight (< 2500 g, *p* = 0.345) and small-for-gestational-age newborns (*p* = 0.469) were similar. The 1- (*p* = 0.191) and 5-min (*p* = 0.174) Apgar points in the groups were similar, and no associations between pregnancy outcomes and surgery technique were detected.

**Conclusions:**

The outcome in pregnancies with an SCT 12 months, postponing pregnancy may not always improve pregnancy outcomes. Therefore, all risks should be weighed when counselling women regarding the optimal time of pregnancy after BS.

## Background

Obesity among women of childbearing age is a growing concern [1]. It increases the risk of adverse pregnancy and delivery outcomes [2–4], including an increased risk for congenital abnormalities, such as heart defects [5]. 

In 2022, the average prepregnancy body mass index (BMI) of parturients in Finland was 26.0 kg/m^2^, and the percentages of overweight (BMI = 25.0–29.9 kg/m^2^) and Class I obese (BMI = 30.0–34.9 kg/m^2^) individuals were 27.7% and 19.5%, respectively [6]. Since 2005, the percentage of parturients living with obesity has increased from 11.0 to 19.5%.

As the most effective treatment for obesity, the number of bariatric surgeries (BSs) is increasing [7], and the majority are performed on women of childbearing age [8]. The effect of BS has traditionally been attributed to either the restriction of stomach size, the malabsorption of nutrients, or both. According to the current understanding, these factors seem to play a smaller role, and a complex interaction of digestive hormones is mainly responsible for weight loss after surgery. The most commonly used techniques are sleeve gastrectomy (SG, a restrictive technique) and Roux-en-Y gastric bypass (RYGB, a combinational technique), but the choice of technique is made individually based on the patient’s BMI and possible comorbidities [9].

Several studies have shown that, by addressing the root causes of obesity-related complications, BS improves pregnancy outcomes and reduces the risk of common pregnancy complications, such as gestational diabetes and hypertensive disorders [10–14]. The post-operative weight loss decreases the release of pro-inflammatory cytokines, lowers maternal insulin resistance, and consequently, the risk of GDM and associated complications [11]. The decrease in inflammatory markers, improved endothelial function and stabilization of metabolic processes reduce the likelihood and severity of hypertensive conditions, particularly during pregnancy [11, 13].

The profound changes in anatomy, nutrient absorption, and metabolism can also compromise the ability to meet the increased nutritional and metabolic demands of pregnancy. Therefore, concerns over an increased risk of low birth weight [15, 16] and preterm delivery after BS have been raised [16]. The outcome of pregnancy can be associated with the surgical technique, and malabsorptive surgeries, such as duodenal switch, may be more prone to an increased risk of small for gestational age (SGA) foetuses than purely restrictive surgeries [17–19]. This is thought to be caused by vitamin and mineral deficiencies resulting from the bypass of the small intestine during surgery. According to the study by Galazis [17], restrictive—rather than malabsorptive—surgeries should be preferred in women wanting a future pregnancy. An increased risk of preterm delivery has been reported in many studies and in a study by Cornthwaite et al., it was reported to be higher after gastric banding (restrictive technique), but comparable after SG and bypass [19]. 

In women of childbearing age, improvements in the menstrual cycle and changes in sex hormone levels can be observed soon after surgery. Spontaneous pregnancy may become possible in previously infertile women [20, 21], and the desire for pregnancy may be high, especially after obesity-related infertility. The correct timing of pregnancy after BS is important, and current recommendations advise postponing pregnancy at least 12–24 months after surgery [22, 23]. This has been justified by concerns over the impact of rapid postoperative weight loss and the body’s active catabolic state during the first 12 months. During this period, the risks of nutritional deficiencies, inadequate weight gain, and metabolic instability are increased and may pose a risk to the well-being of the mother and the foetus.

Also in Finland, a surgery-to-conception time (SCT) of at least one year is recommended [22, 23]. The weight should be stable, and the regular use of dietary supplements (multivitamins, B_12_-vitamins, iron, calcium + vitamin D) is advisable before pregnancy begins. The consensus concerning the importance of vitamin supplementation is broad, but minor differences can be seen in the recommended dosages across different institutions [24, 25]. Three months before a desired pregnancy, a folic acid supplement should be added, and vitamin A supplementation should be changed to beta carotene.

The rationale behind these current recommendations concerning pregnancy timing has been questioned by some previous studies, which have found no support for them [26], neither after laparoscopic SG [27] nor RYGB [28]. Therefore, the timing of pregnancy requires a comprehensive evaluation, with the understanding that the possible weight regain [29] and advancing maternal age reduce fertility and increase pregnancy risk if women are advised to wait too long after BS [30].

The current retrospective study aimed to analyse the possible differences in maternal and foetal outcomes between pregnancies with an SCT of less than 12 months and those with an SCT of more than 12 months.

## Methods

All women in the Helsinki-Uusimaa Hospital district with a delivery between 2010 and 2022 and a BS prior to pregnancy were included in this register-based cohort study. Patients with a previous BS were identified from the Hospital Register and Medical Birth Register were queried for data on pregnancies, deliveries and neonatal outcomes. The data were then merged according to their personal identification number.

The SCT, maternal characteristics at surgery (age, weight, BMI) and type of surgery were analysed. Maternal early pregnancy characteristics (age, prepregnancy weight, prepregnancy BMI, parity, primiparity, prepregnancy diabetes mellitus (DM) type II), pregnancy complications (GDM, preeclampsia, foetal demise), delivery outcomes (gestational weeks (gw) at delivery, preterm and post-term deliveries, delivery induction, caesarean delivery) and newborn outcomes (birth weight, birth weight standard deviation (SD), low birth weight, SGA, 1- and 5-min Apgar points) were collected and analysed. In the case of a twin pregnancy, only the outcomes of the A-foetus were included in the study. The type of BS and associated outcomes were also analysed.

The time of conception was assessed based on the date and gestational age at birth. To diagnose GDM, fasting blood glucose and one-hour postprandial values were monitored for one week between 24 and 28 weeks of gestation (gw). Two 24-hour monitoring periods with fasting and one-hour postprandial values at all meals (breakfast, lunch, dinner, and snacks) were included. A regular glucose tolerance test is avoided after BS as altered metabolism can lead to hypoglycemia [15] following an early increase in glucose levels [12] (postprandial dumping syndrome). Plasma glucose fasting and one-hour postprandial values of ≥ 5.5 and ≥ 7.8 mmol/L, respectively, were used to diagnose GDM. Preeclampsia was diagnosed as a blood pressure > 140/90 mmHg after 20 + 0 gw, combined with at least one of the following: proteinuria, elevated liver or kidney values, thrombocytopenia, neurological symptoms or foetal growth restriction. Pre- and post-term deliveries were assessed as deliveries before 37 + 0 and after 41 + 5 gw, and low birth weight and SGA were diagnosed as a weight < 2500 g and < -1.3 SD (= 10th percentile) at birth.

All pregnancies were monitored by either a specialist in obstetrics and gynaecology or maternal-foetal medicine. Laboratory tests (ionised calcium, ferritin, C-reactive protein, complete blood count, folate, B_12_-vitamin, albumin and vitamin D) were performed every trimester, and an obstetric appointment was carried out at 28, and 36 gw. The method and timing of delivery were based on obstetric indications.

The Helsinki University Hospital Review Board approved the study and its plan. Ethics committee approval was not needed and based on national legislation (Medical Research Act 488/1999), the requirement for written informed consent was waived for this retrospective study.

Statistical analyses were performed using SAS version 9.4 software (SAS Institute, Cary, NC, USA), and *p* < 0.05 was considered statistically significant. Continuous and categorical variables were compared using the Mann–Whitney U test and the *X* [2] test or Fisher’s exact test, respectively. Linear regression was used to analyse the linear relationships between variables.

## Results

Overall, 113 women gave birth after BS, and 17 of them (15%) had conceived during the first postoperative year. The mean SCT among all women was 34 ± 25 months (2.8 ± 1.2 years), with the shortest and longest SCTs being 3.2 months and 9.5 years, respectively. The mean SCTs among those who conceived during (Group 1, *n* = 17) and after (Group 2, *n* = 96) the first postoperative year were 8.0 ± 2.7 and 39.0 ± 24.3 months (3.3 ± 2.0 years), respectively. The pregnancies in Group 2 included one twin pregnancy.

The mean age (*p* = 0.316), preoperative weight (*p* = 0.718), and BMI (*p* = 0.114) of women at the time of BS were similar among the groups. The surgical technique was available for 77 patients (68.1%), and in all patients, either RYGB or SG was used. The incidence of techniques (RYGB or SG) in the groups was similar (*p* = 0.646) (Table 1).

**Table 1:** The surgery-to-conception time and characteristics of women at surgery

	Group 1 (*n* = 17)	Group 2 (*n* = 96)	*p*-value
Surgery-to-conception time (months)	8.0 ± 2.7	39.0 ± 24.3	< 0.001
Age at surgery (years)	30.6 ± 5.9	31.9 ± 4.8	0.316
Preoperative weight (kg)	127.3 ± 21.6	129.4 ± 21.7	0.718
Preoperative BMI (kg/m^2^)	37.4 ± 6.4	40.6 ± 7.6	0.114
Roux-en-Y bypass surgery*	6 (46.2)	34 (53.8)	0.646
Sleeve gastrectomy	7 (53.1)	30 (46.9)	0.646

Values are presented as mean (SD) or number (%)

The method of surgery missing in 36 (31.9%)) cases

*Including one case of one-anastomosis gastric bypass

At the time of pregnancy, the women in Group 1 were significantly younger (*p* = 0.005), but no difference was detected in the mean prepregnancy weight (*p* = 0.961) or BMI (*p* = 0.567). The mean parity of the women in the groups was similar (*p* = 0.995), as was the incidence of primiparity (*p* = 0.407). There was no difference in the incidence of non-insulin-treated DM II among women (*p* = 0.909). In Group 2, GDM was significantly more common (*p* = 0.039), but there was no difference in the incidence of pre-eclampsia (*p* = 1.00) or foetal death (*p* = 1.00). Only one case of foetal demise at 35 gw was found in Group 2.

The mean gestational age at delivery in the groups was similar (*p* = 0.206), and no difference was detected in the incidence of preterm (*p* = 0.645) or post-term deliveries (*p* = 1.00). A significant difference was detected in the incidence of induced deliveries (*p* < 0.001). Compared with the induction incidence of 59.4% in Group 2, only one delivery (5.9%) in Group 1 was induced. The number of all caesarean deliveries (*p* = 1.00) and unplanned caesarean (*p* = 0.462) did not differ (Table 2).

**Table 2:** Pregnancy and delivery outcomes

	Group 1 (n = 17)	Group 2 (n = 96)	*p-*value
**Pregnancy outcomes**			
Early pregnancy age (years)	31.2 ± 5.9	35.1 ± 5.0	0.005
Prepregnancy weight (kg)*	95.5 ± 22.8	95.8 ± 18.2	0.961
Prepregnancy BMI (kg/m^2^)*	37.4 ± 6.4	40.6 ± 7.6	0.567
Parity	1.3 ± 1.6	1.3 ± 1.4	0.995
Primiparity	8 (47.1)	35 (36.5)	0.407
Diabetes mellitus type II	1 (5.9)	5 (5.2)	0.909
Gestational diabetes mellitus	1 (5.9)	29 (30.2)	0.039
Pre-eclampsia	0 (0)	5 (5.2)	1.00
Foetal demise	0 (0)	1 (1.0)	1.00
**Delivery outcomes**			
Gestational age at delivery (gw)	38.1 ± 3.9	39.4 ± 1.7	0.206
Preterm delivery	2 (11.8)	8 (8.3)	0.645
Post-term deliveries	0 (0)	4 (4.2)	1.00
Induction of delivery	1 (5.9)	57 (59.4)	< 0.001
Caesarean delivery (all)	3 (17.6)	20 (20.8)	1.00
Caesarean deliveries (unplanned)	1 (5.9)	14 (14.6)	0.462
**Newborn outcomes**			
Birth weight (g)	3053.8 ± 843.1	3387.7 ± 555.1	0.038
Birth weight (SD)	-0.37 ± 1.0	-0.33 ± 1.1	0.883
Low birth weight	2 (11.8)	6 (6.3)	0.345
Small for gestational age	4 (23.5)	14 (14.6)	0.469
Apgar 1 min	8.00 ± 2.2	8.76 ± 1.3	0.191
Apgar 5 min	8.57 ± 1.9	9.33 ± 1.4	0.174

Values are presented as mean (± SD) or number (%). gw=gestational weeks, SD=standard deviation

*Self-reported prepregnancy weight at the first outpatient clinic appointment

The mean birth weight of the newborns in Group 1 was significantly lower (*p* = 0.038), but there was no difference in the mean birth weight SD (*p* = 0.883). The incidence of low birth weight (*p* = 0.345) and SGA (*p* = 0.469) in the groups was similar. Both cases (2/2) of low birth weight in Group 1 and 4/6 cases in Group 2 were associated with preterm delivery. In five cases (5/8), the newborn with a low birth weight was also SGA, all of whom were in Group 2. The Apgar scores at 1 (*p* = 0.191) and 5 min (*p* = 0.174) in the groups were similar (Table 2). When a simple linear regression model was used to test whether SCT could predict either birth weight or gestational age at birth, the result with our sample size was nonsignificant in both cases (*p* = 0.300 for birth weight, *p* = 0.117 for gestational age at birth).

Delivery inductions in Group 2 were further analysed. Among the groups, no difference was detected in the mean SCT (*p* = 0.154). The mean age (*p* = 0.122) of the women was similar, but the mean prepregnancy weight (*p* = 0.004) and BMI (*p* = 0.004) were significantly higher among those with induced deliveries. The mean parity of women (*p* = 0.737) and incidence of primiparity (*p* = 0.338) were similar among induced and spontaneous deliveries. There was no difference in the incidence of non-insulin-treated prepregnancy type II DM (*p* = 0.645), but the GDM incidence (*p* = 0.030) was significantly higher among those with induced deliveries. The incidences of pre-eclampsia (*p* = 0.645) and cholestasis of pregnancy (*p* = 0.512) in the groups were similar. There was no difference in the mean gestational age at delivery (*p* = 0.310), and the incidence of post-term pregnancies and the need for unplanned caesarean delivery were similar among the groups (*p* = 0.388).

The mean newborn weight (*p* = 0.211), mean birth weight SD (*p* = 0.153), low birth weight (*p* = 1.00) and SGA incidences (*p* = 0.275), as well as the 1- (*p* = 0.564) and 5-minute (*p* = 0.799) Apgar scores were similar between the groups. There was no difference in the surgical technique used (*p* = 0.610) between induced and spontaneous deliveries (Table 3). Compared with Group 1, the odds ratio for delivery induction in Group 2 was 23.4 (95% CI, 2.98–183.6).

**Table 3:** The comparison of induced and noninduced deliveries in Group 2

	Induction of delivery (*n* = 57)	No induction of delivery (*n* = 39)	*p*-value
**Pregnancy and delivery**			
Surgery-to-conception time (months)	42.0 ± 26.9	34.8 ± 19.6	0.154
Early pregnancy age (years)	35.8 ± 5.4	34.2 ± 4.3	0.122
Prepregnancy weight (kg)*	99.95 ± 16.9	88.82 ± 18.4	0.004
Prepregnancy BMI (kg/m^2^)*	36.03 ± 6.25	32.09 ± 5.78	0.004
Parity	1.33 ± 1.2	1.23 ± 1.8	0.737
Primiparity	23 (40.4)	12 (30.8)	0.338
Gestational diabetes mellitus	22 (38.6)	7 (17.9)	0.030
Diabetes mellitus type II	4 (7.0)	1 (2.6)	0.645
Pre-eclampsia	4 (7.0)	1 (2.6)	0.645
Cholestasis of pregnancy	2 (3.5)	0 (0)	0.512
Gestational age at birth (gw)	39.6 ± 1.46	39.2 ± 1.97	0.310
Post-term deliveries	2 (3.5)	2 (5.1)	1.00
Caesarean delivery (unplanned)	10 (17.5)	4 (10.3)	0.388
**Newborn**			
Birth weight (g)	3446.5 ± 580.5	3301.6 ± 510.9	0.211
Birth weight (SD)	-0.22 ± 1.1	-0.52 ± 0.9	0.153
Low birth weight	4 (7.0)	2 (5.1)	1.00
Small for gestational age	7 (12.3)	8 (20.5)	0.275
Apgar 1 min	8.82 ± 1.39	8.67 ± 1.10	0.564
Apgar 5 min	9.36 ± 1.55	9.28 ± 0.99	0.799
**Surgery**			
Roux-en-Y gastric bypass surgery	22 (56.4)	12 (48.0)	0.610
Sleeve gastrectomy	17 (43.6)	13 (52.0)	0.610

Values are presented as mean (± SD) or number (%). gw=gestational weeks, SD=standard deviation

*Self-reported prepregnancy weight at the first outpatient clinic appointment

Finally, the associations of surgical technique with pregnancy and delivery outcomes were analysed. No difference was detected in the mean age at surgery (*p* = 0.829), and the mean preoperative weight (*p* = 0.301) and BMI (*p* = 0.466) of the women were similar. The incidence of GDM (*p* = 0.128) was similar across the groups. There was no difference in the mean gestational age at delivery, and the incidence of preterm deliveries (*p* = 0.907), post-term deliveries (*p* = 0.106), induced deliveries (*p* = 0.311), and caesarean deliveries (*p* = 0.129) in the groups were similar. There was no association between surgery technique and the incidence of low newborn weight (*p* = 0.705) or SGA (*p* = 1.00) (Table 4).

**Table 4:** The comparison of surgery techniques, pregnancy and newborn outcomes

	RYGB (*n* = 40)	SG (*n* = 37)	*p*-value
**Surgery**			
Age at surgery (years)	34.0 ± 5.5	33.7 ± 5.0	0.829
Preoperative weight (kg)	130.2 ± 24.4	124.9 ± 19.2	0.301
Preoperative BMI (kg/m^2^)	40.3 ± 7.4	39.1 ± 7.3	0.466
**Pregnancy and delivery**			
Gestational diabetes mellitus	15 (37.5)	8 (21.6)	0.128
Gestational age at delivery (gw)	273.2 ± 10.9	276.0 ± 15.9	0.168
Preterm delivery	4 (10.0)	4 (10.8)	0.907
Post-term delivery	0 (0)	3 (8.1)	0.106
Caesarean delivery (all)	7 (17.5)	12 (32.4)	0.129
Caesarean delivery (unplanned)	4 (10.0)	8 (18.9)	0.213
Induction of delivery	23 (57.5)	17 (45.9)	0.311
**Newborn**			
Birthweight (g)	3307.3 ± 535.2	3329.0 ± 643.1	0.873
Low birth weight	3 (7.5)	4 (10.8)	0.705
Small for gestational age	7 (17.5)	6 (16.2)	1.00

Values are presented as mean (SD) or number (%)

RYGB = Roux-en-Y gastric bypass surgery, SG = sleeve gastrectomy, gw=gestational weeks

## Discussion

The timing of conception after bariatric surgery plays a crucial role in determining whether the benefits of the surgery outweigh the potential risks. In general, women are advised to wait until the phase of rapid weight loss has ended and a more stable metabolic state is achieved.

In the present retrospective study, the timing of pregnancies after BS varied from 3.2 months to 9.5 years. However, 63% of women (71/113) conceived within three years after surgery. The overall outcome in pregnancies conceived during the first postoperative year was good: These women were significantly younger, and the incidences of GDM and delivery induction were significantly lower. The mean newborn weight was slightly lower, but the incidence of preterm delivery did not increase.

In the present study, women with SCTs of less than or more than 12 months had similar characteristics at their BS. No difference was detected in the surgical technique used. As reported in our previous study [31], the incidence of pre-eclampsia after BS was low, with no difference between groups. The studies by Sheiner et al. [26] and Dao et al. [32] reported concordant results when comparing pregnancies conceived during and after the first postoperative year.

In our previous work, we reported that the risk for GDM is still significantly increased after BS [31]. This is most likely associated with the residual obesity after BS. However, compared with the 20% incidence of GDM in Finland [33], the incidence of GDM in Group 1 was extremely low (5.9%). We acknowledge that our small sample size is prone to bias; however, a Swedish study by Johansson et al. [9] reported a post-BS GDM incidence of 1.9% among women with a median surgery-to-delivery interval of 1.8 years (interquartile range, 1.4–2.5) and a mean presurgery BMI of 43.7 kg/m^2^. The exact diagnostic criteria for GDM in the study were not reported. Different results were reported in a study by Froylich et al., [27] which showed no difference in the incidence of GDM (one-hour blood glucose level ≥ 7.8 mmol/L after a 50 g dose of oral glucose) among pregnancies that were conceived less than 12 months, 12–24 months or more than 24 months after laparoscopic SG. Additionally, a study by Sheiner et al. [26] indicated a nonsignificant difference in the incidence of GDM among women who conceived during (*n* = 104) or after (*n* = 385) the first post-BS year (10.5% vs. 7.3%, *p* = 0.159). In their study, GDM was assessed as glucose intolerance first recognised in the ongoing pregnancy. The difference in BMI before pregnancy and after delivery was not significant. Differences in the diagnostic criteria for GDM may explain these conflicting results, but they also make comparisons among studies difficult. However, we believe that our protocol, which involved one week of glucose level monitoring, provides reliable information on glucose tolerance during pregnancy.

The study results of Shah et al. [34]. may explain the significant difference in GDM incidence between our study groups; they reported that the glycaemic control of obese patients significantly improved during the first post-BS year. Rapid weight loss is assumed to play an important role, but associated hormonal factors are also important [35]. The long-term data of post-BS patients revealed evidence of hyperglycaemia recurrence years after surgery [34]. Additionally, the risk of GDM linearly increases with increasing maternal age [36]. 

An increased risk of preterm delivery after BS has been reported in previous studies [17, 31, 37]. Compared with the national incidence of preterm birth (5%),^6^ the incidence in both study groups (11.8% and 8.3%) and after both surgery techniques (10.0% and 10.8%) was high. Overall, 10 cases of preterm birth were detected, with most of them (7/10) being late preterm deliveries (> 34 gw), including three cases at 36 gw.

In our previous study, we reported an increased risk of planned and unplanned caesarean delivery after BS [31]. However, the present study revealed no association with SCT. Similarly, the study by Sheiner et al. [26] detected no difference in pregnancies with an SCT of less (mean 8.0 months) or more than 12 months (mean 56.7 months), but a significantly higher risk associated with an SCT of more than 24 months was reported by Froylich et al. [27]

An extremely high incidence (59.4%) of delivery inductions was detected in Group 2. The mean prepregnancy weight and BMI of women with induced delivery were significantly higher, and the most common indication for induction (38.6%) was GDM. The study by Sheiner et al. [26] reported opposite results and detected no difference in delivery induction between pregnancies conceived during and after the first postoperative year. Neither did they detect any difference in maternal age or GDM incidence, which may explain the difference compared with our results. An increased risk of delivery induction after BS was reported in a study by Abenheim et al., [38] which compared post-BS women to morbidly obese, nonoperated pregnant women.

Although the small sample size in our study is prone to bias, the high incidence of inductions must be considered a sign of increased concern among these pregnancies. Because all pregnancies were followed in the same hospital, this cannot be explained by different guidelines for delivery induction. Even though we did not detect induction-associated differences in the incidence of unplanned caesarean deliveries or newborn outcomes, delivery induction means intervening in the normal course of pregnancy and is not without risk.

Previous studies have reported nutritional deficiencies and increased risk of low birth weight after BS [39–42]. Compared with the national data on mean newborn weight (3498 g) and the incidence of low birth weight (4.2%),^6^ the mean birth weight was lower, and the incidence of low birth weight was higher in both groups. In 75% (6/8) of the cases, low birth weight was associated with preterm delivery. The mean birth weight was significantly lower in Group 1, which included two preterm deliveries (at 25 gw and 32 gw) with low birth weight newborns (730 g and 2010 g). However, no difference was detected in the mean SD of birth weight (-0.37 and − 0.33).

Previous results concerning the association between SCT and foetal growth are conflicting. Norgaard et al. [41] reported no correlation between the incidence of SGA and SCT, suggesting that the risk of SGA is associated with a history of BS itself rather than with SCT. In contrast, the study by Parent et al. [42] reported a greater risk for SGA up to three years after BS and a greater risk of prematurity and neonatal intensive care admission with an SCT of less than two years. Accordingly, an SCT of at least 24 months was suggested in a study by Carreira et al. [43] to reduce the risk of SGA. In the present study, the incidence of SGA (< 10th percentile) was not significantly higher in patients with an SCT less than 12 months (23.5% vs. 14.6%). We were unable to show any association between newborn weight and surgery technique, but previous studies reported lower newborn weights after RYGB bypass than after SG [18, 19, 44]. A larger sample size may be necessary to study the impact of different surgery types on foetal growth.

Although guidelines [22] and consensus recommendations [23] still advise delaying pregnancy for at least 12–18 months after BS, some studies have reported the safety of conceiving within one year [26, 38]. However, relatively small study populations have prevented strong conclusions from being drawn [45–47]. The optimal timing of pregnancy involves balancing the risks of short SCT and risks associated with advancing age and possible weight regain as time after surgery increases. If contraception is not carefully planned, an unplanned pregnancy is also possible when the menstrual cycle returns [48]. 

We acknowledge that the present study has several limitations. The small number of pregnancies with an SCT of less than 12 months is prone to bias. Because women are advised to wait at least one year before conceiving after BS, it is reasonable to assume that the number of these patients is small. A larger study population would have allowed us to study pregnancy and delivery outcomes and outcome trends more reliably. Missing data on surgical techniques may also have caused some bias in the results, which can be considered another weakness of the current study.

High-quality register-based data from the Finnish Institute of Health and Welfare can be considered a strength of the present study. Additionally, the follow-up of pregnancies and treatment of deliveries in the same hospital and with the same principles can be regarded as another strength of the study.

## Conclusion

The present study compared pregnancies conceived during and after the first year post-BS. Women with a SCT of more than 12 months were older, but otherwise, no differences were detected in the characteristics of women at surgery.

Our study reinforces prior research showing an increased risk of preterm delivery after BS. However, most cases in this study were late-preterm and unrelated to the SCT. The incidence of low birth weight was also increased in both groups, but it was predominantly linked to preterm deliveries. Unlike many previous studies, we also analysed newborn weight SDs and found no differences between the groups. We conclude that the mean gestational age (38 weeks) and birth weight (3053 g) in deliveries with an SCT < 12 months can be considered reassuring.

The association between GDM, BS, and SCT remains complex, at least partly due to varying diagnostic criteria for GDM. Unlike most previous studies, we identified a very low incidence of GDM during the first year after BS along with a significant difference between groups This is likely explained by higher maternal age and the recurrence of hyperglycemia among pregnancies with SCT > 12 months. The incidence of labour inductions was significantly higher among pregnancies with a SCT > 12 months, predominantly driven by GDM.

We conclude that a comprehensive evaluation of maternal health and obstetric history should be performed when women are advised concerning the optimal time for pregnancy after BS. While counselling patients on all possible risks associated with a short SCT, the risks of postponing pregnancy should also be weighed. Conceiving during the first postoperative year can be considered safe if it is assumed that postponing the pregnancy may not improve the overall prognosis of the pregnancy and delivery.

## Data Availability

The data that support the findings of this study are available from the corresponding author upon reasonable request.

## Abbreviations

BMI: Body mass index
BS: Bariatric surgery
SG: Sleeve gastrectomy
RYGB: Roux-en-Y gastric bypass
SGA: Small for gestational age
SCT: Surgery-to-conception time
DM: Diabetes mellitus
GDM: Gestational diabetes mellitus
SD: Standard deviation
